# Author Correction: Infection-induced type I interferons critically modulate the homeostasis and function of CD8^+^ naïve T cells

**DOI:** 10.1038/s41467-024-44731-3

**Published:** 2024-01-11

**Authors:** Mladen Jergović, Christopher P. Coplen, Jennifer L. Uhrlaub, David G. Besselsen, Shu Cheng, Megan J. Smithey, Janko Nikolich-Žugich

**Affiliations:** 1 Department of Immunobiology and the University of Arizona Center on Aging, University of Arizona College of Medicine, Tucson, AZ USA; 2 University Animal Care, University of Arizona, Tucson, AZ USA; 3 Department of Medicine, University of Arizona College of Medicine, Tucson, AZ USA; 4 Present Address: Vir, Inc., San Francisco, CA USA

**Keywords:** Interferons, Infection, Cytotoxic T cells, Signal transduction

Correction to: *Nature Communications* 10.1038/s41467-021-25645-w, published online 06 September 2021

In the published version of the manuscript, the two panels in Figure 8A were inadvertently shown as duplicates in Figure 8B. This has now been corrected in both the PDF and HTML versions of the Article.

