# Supplementary material for: Infection-induced type I interferons critically modulate the homeostasis and function of CD8+ naïve T cells
Source: Nat Commun. 2021 Sep 6;12:5303. doi: 10.1038/s41467-021-25645-w (PMC8421345; doi:10.1038/s41467-021-25645-w)
Supplement: Supplementary file 1 — Supplementary Information [file 41467_2021_25645_MOESM1_ESM.pdf]

Supplemental Information

Infection-induced type I interferons critically modulate the homeostasis and function of CD8$^+$ naïve T cells

Mladen Jergovic[1], Christopher P. Coplen[1], Jennifer L. Uhrlaub[1], David Besselsen[2], Shu Cheng[3], Megan J. Smithey[1,4], Janko Nikolich-Žugich[1,*]

[1] Department of Immunobiology and the University of Arizona Center on Aging, University of Arizona College of Medicine – Tucson, Tucson, AZ, USA;
[2] University Animal Care, University of Arizona, Tucson, USA;
[3] Department of Medicine, University of Arizona College of Medicine-Tucson, Tucson, USA
[4] - Present address: Vir, Inc., San Francisco, CA, USA.

[*]- To whom correspondence should be addressed at nikolich@email.arizona.edu or P.O. Box 249221, 1501 N. Campbell Ave. Tucson, AZ 85724, USA.

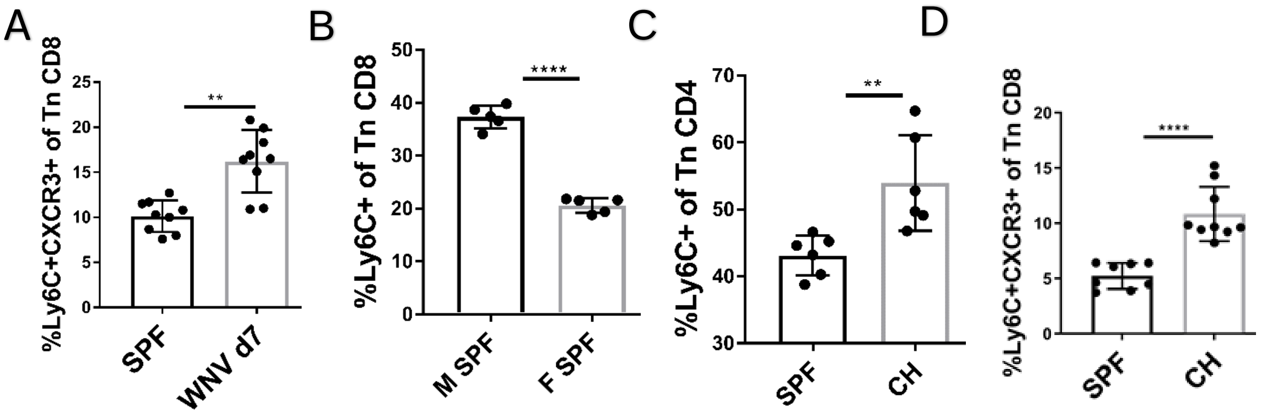

Supplemental Figure 1. Percentage of Ly6C+ and Ly6C+CXCR3+ cells. A) C57BL/6 mice (n=9) were infected with 1000 PFU WNV virus by footpad injection. Mice were bled retro-orbitally at day 7 p.i. and percentage of Ly6C^{+}CXCR3^{+} Tn CD8s was measured (data presented as mean ± sd, **p=0.0012).B) percentage of Ly6C^{+} on CD8^{+} Tn cells of male and female C57/BL6 mice under SPF conditions (n=5, data presented as mean ± sd, ****p<0.0001)**. C)** Percentage of Ly6C^{+} on CD4^{+} Tn cells in SPF and CH mice (n=6, data presented as mean ± sd, **p=0.0022)**. D)** percentage of Ly6C^{+}CXCR3^{+} Tn CD8s was measured in blood of SPF (n=8) and CH (n=9) mice (data presented as mean ± sd, ****p<0.0001). A,C, D Two-tailed Mann-Whitney U test, B two tailed Student's t-test. A,B data from one experiment, C data representative of two independent experiments, D data pooled from two independent experiments.

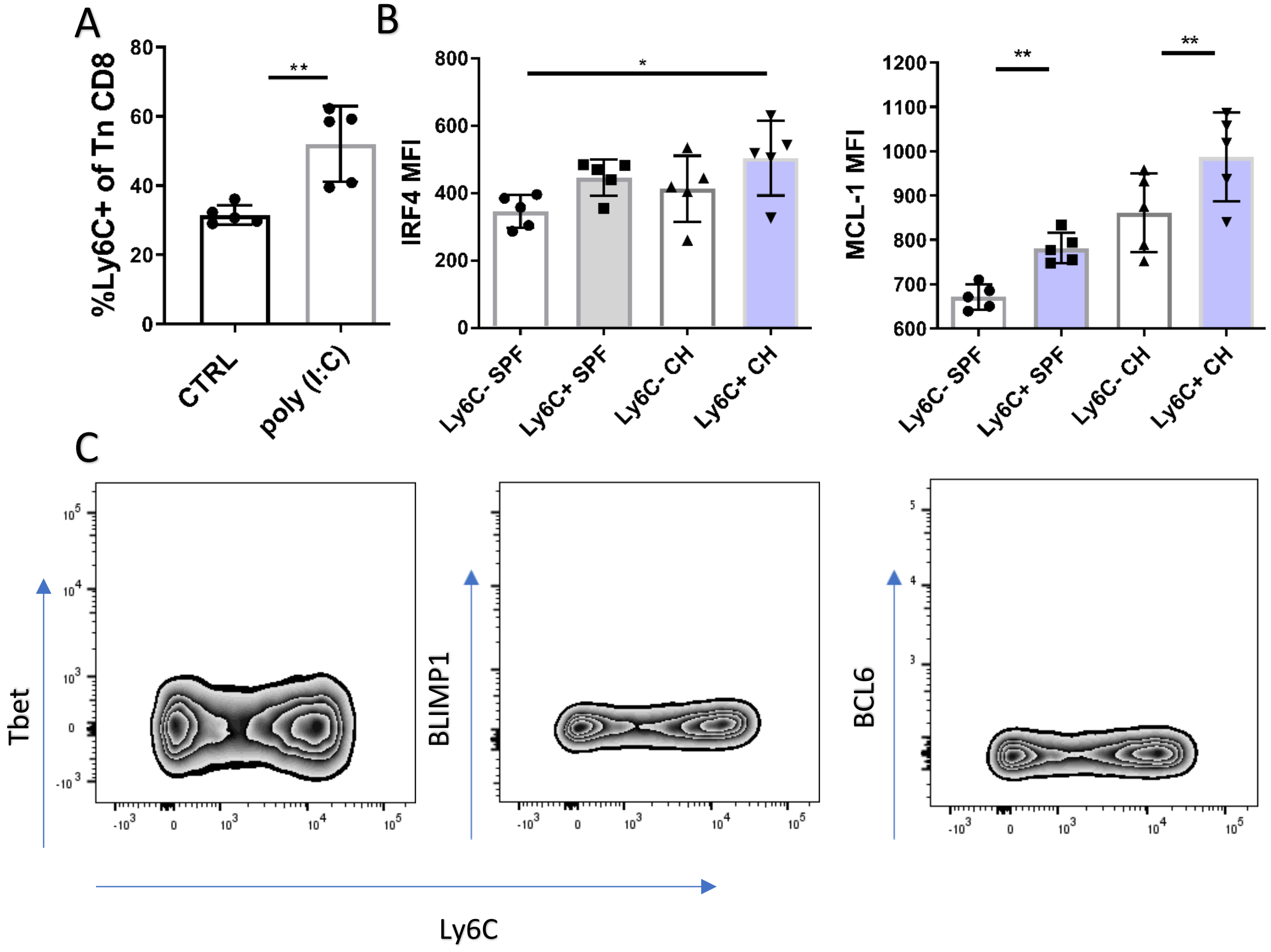

Supplemental Figure 2. Expression of various transcription factors on Ly6C$^+$ Tn cells. A) SPF mice were treated with 100 µg poly(I:C) intraperitoneal injection. 48h hours later mice were bled retroorbitally and expression of Ly6C on CD8$^+$ Tn was analyzed (n=5 mice per group, data presented as mean ± sd, **p=0.0037). **B)** Expression of MCL-1 and IRF4 on Ly6C±CD8$^+$ Tn from SPF and CH (n=5 mice per group, data presented as mean ± sd, left panel *p=0.0235, right panel **p=0.0035 (left), **p=0.0017 (right)). **C)** No expression/difference in Tbet, BCL-6 and BLIMP-1 levels on Ly6C$^+$CD8$^+$ Tn. A two tailed Student's t-test., B one-way ANOVA with Sidak post hoc correction. A,B data representative of two independent experiments with n=5.

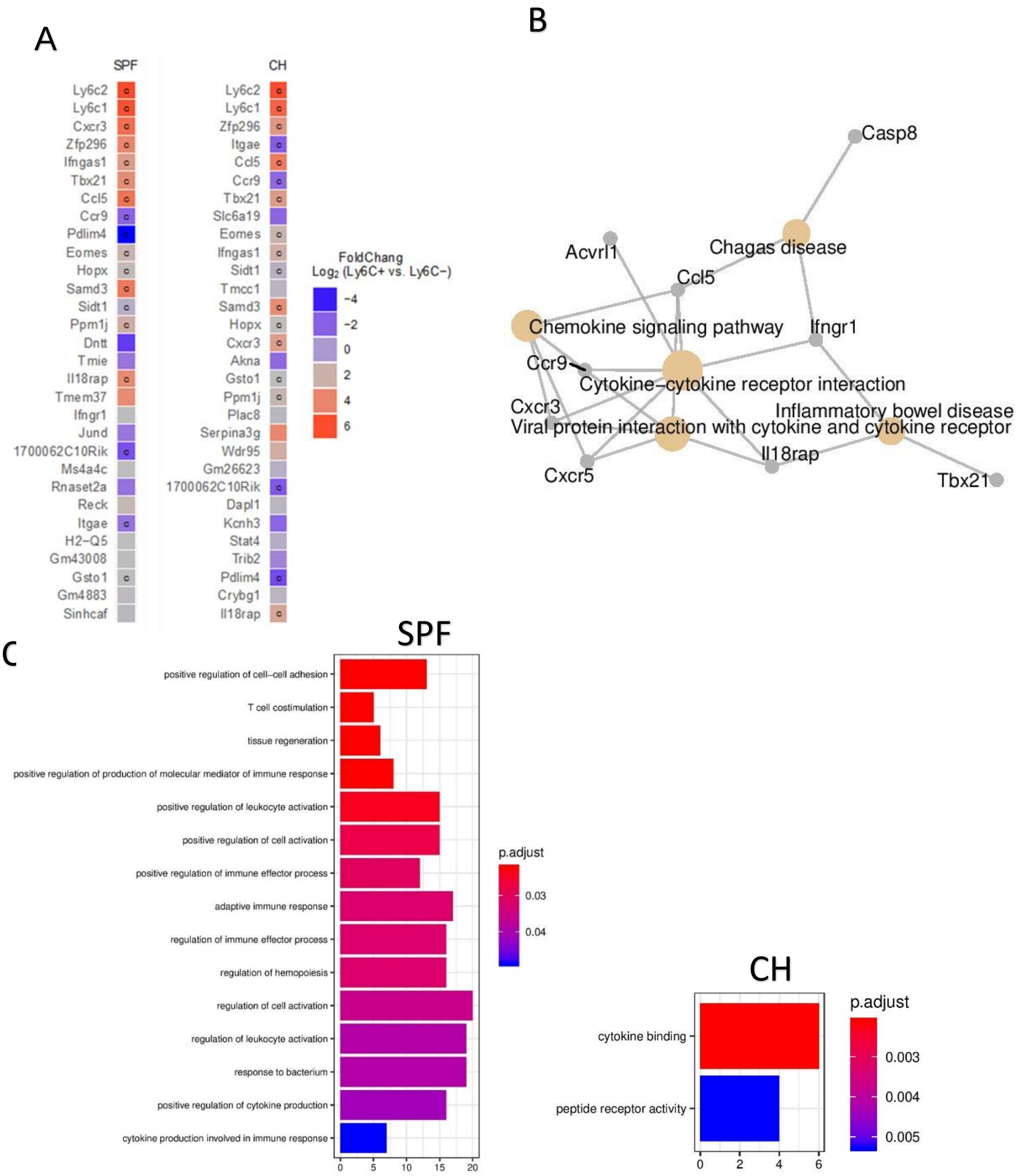

Supplemental Figure 3. Gene list and pathway analysis. **A)** Detailed gene list and expression values **B)** Enriched pathways (orange nodes, $q < 0.1$) identified by KEGG gene set enrichment analysis using overlapped 50 significant genes. Network depicts the linkages of genes and KEGG pathways. The 30 most significant genes and functional enrichment using all significantly differentially expressed genes associated with Ly6C from the SPF and CH mice. **C)** Enriched terms (level 4, $q < 0.1$) identified by Gene Ontology enrichment analysis in SPF and CH mice. Data is from one experiment with nN=4 mice/group

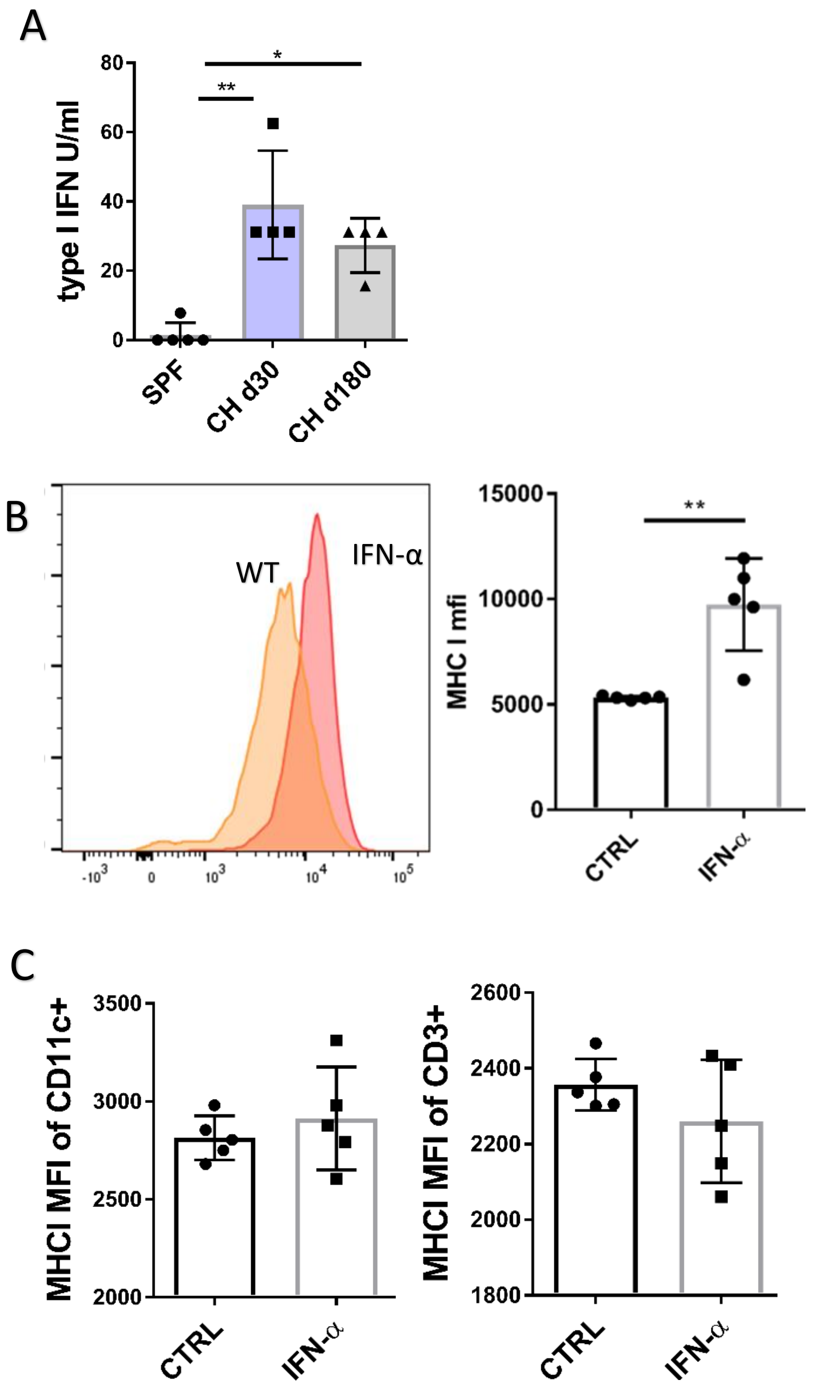

Supplemental Figure 4. **IFN-I levels and MHC-I expression A)** Serum levels of IFN-I were measured by bioassay using IFN responsive cells IFN-responsive L929 cells and Indiana vesiculovirus (SPF mice n=5. CH mice n=4, data presented as mean ± sd, *p=0.0429, **p=0.0051) **B)** LN pools (inguinal, brachial, cervical) were harvested from IFN-α treated mice and control wt mice. LN were digested with GentleMACS and Liberase^{TL} enzyme mixture. FRC were identified as CD45/Ter119 negative gp38+ cells and their expression of MHCI was measured (n=5 mice per group, data presented as mean ± sd, **p=0.0020).**C)** C57BL/6 mice (n=5) were treated with 0.75 µg/mouse IFN-α. After 48 hr LN pool were harvested and expression of MHCI was measured on $CD3^+$ cells and $CD3^-CD11c^+$ cells. A Kruskal-Wallis test with Dunn's correction, B-D unpaired Student's t-test. Data are from one experiment.

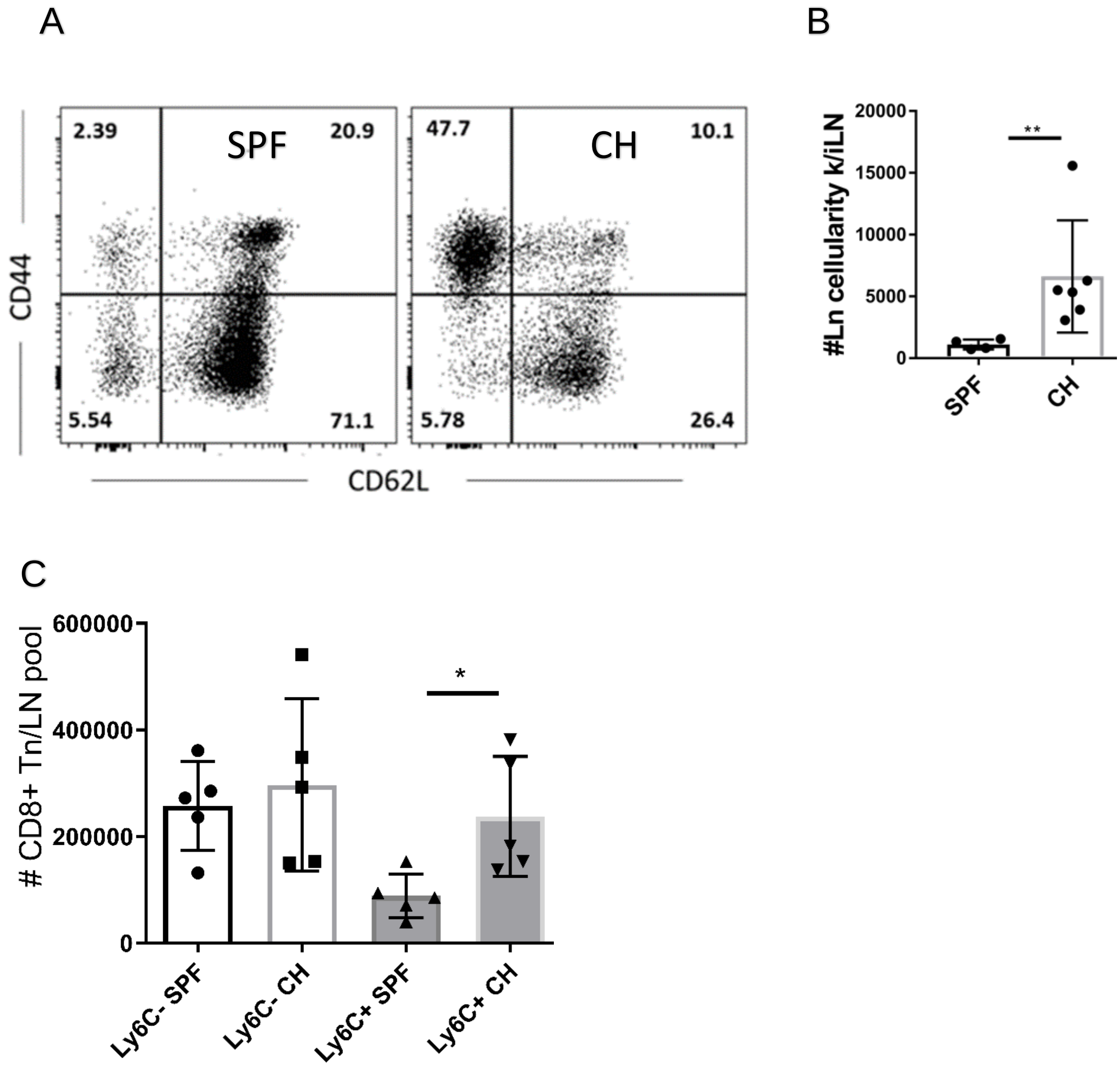

Supplemental Figure 5. Increased lymph node cellularity in cohoused mice A) Expression of CD44 and CD62L on $CD8^+$ T cells from SPF and CH mice. **B)** cellularity of LN from SPF and CH mice measured by Hemavet, veterinary hematology system (SPF n=4mice, CH n=6 mice, data presented as mean ± sd, *p=0.0095) **C)** absolute numbers of CD8 Tn cells in LN pools of SPF and CH mice (n=5 mice per group, data presented as mean ± sd, *p=0.0236). B Mann-Whitney U test, C unpaired Student's t-test. A-C Data representative of two independent experiments.

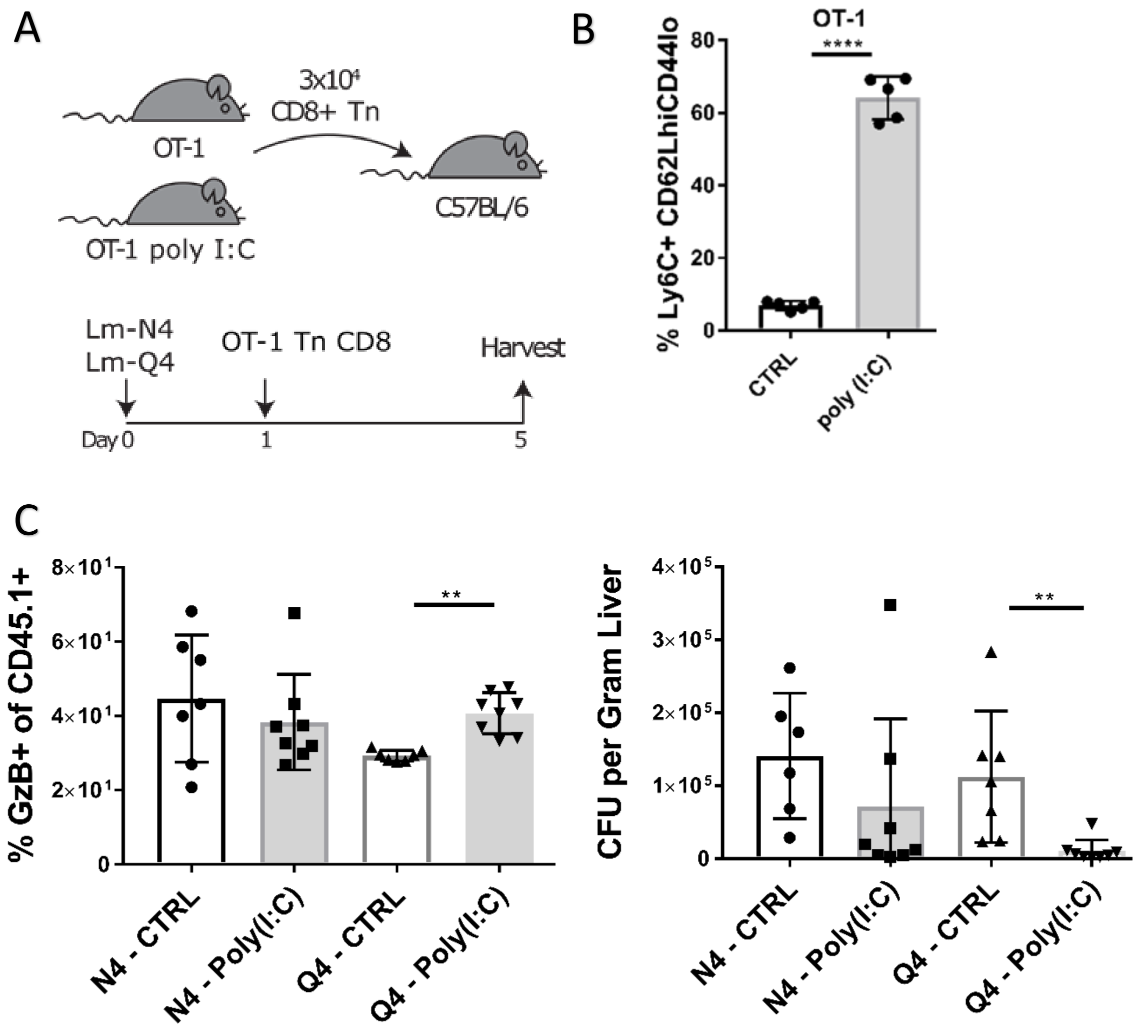

Supplemental Figure 6. Enhanced effector function of Tn CD8s from poly(I:C) pretreated OT-1 mice A) OT-1 transgenic mice were treated 100 µg poly(I:C) intraperitoneal injection prior to CD8 Tn transfer into Lm infected C57/BL6 mice (scheme left). **B)** Pretreatment resulted in upregulation of Ly6C (right panel) after 48h (n=5 mice per group, data presented as mean ± sd, ****p < 0.0001). **C)** Tn CD8s from control and poly(I:C) pretreated mice were transferred into Lm-N4 and LmQ4 mice at d1 p.i. On day 5 cells from pretreated mice exhibited higher GzB expression in spleen (left panel) and lower bacterial burden in liver (right panel) (n=7 ctrl mice, n=8 poly (I:C treated mice, data presented as mean ± sd, **p=0.0012). B unpaired Students-t test. C Mann-Whitney U test between mice groups receiving same APL. Data from one experiment.

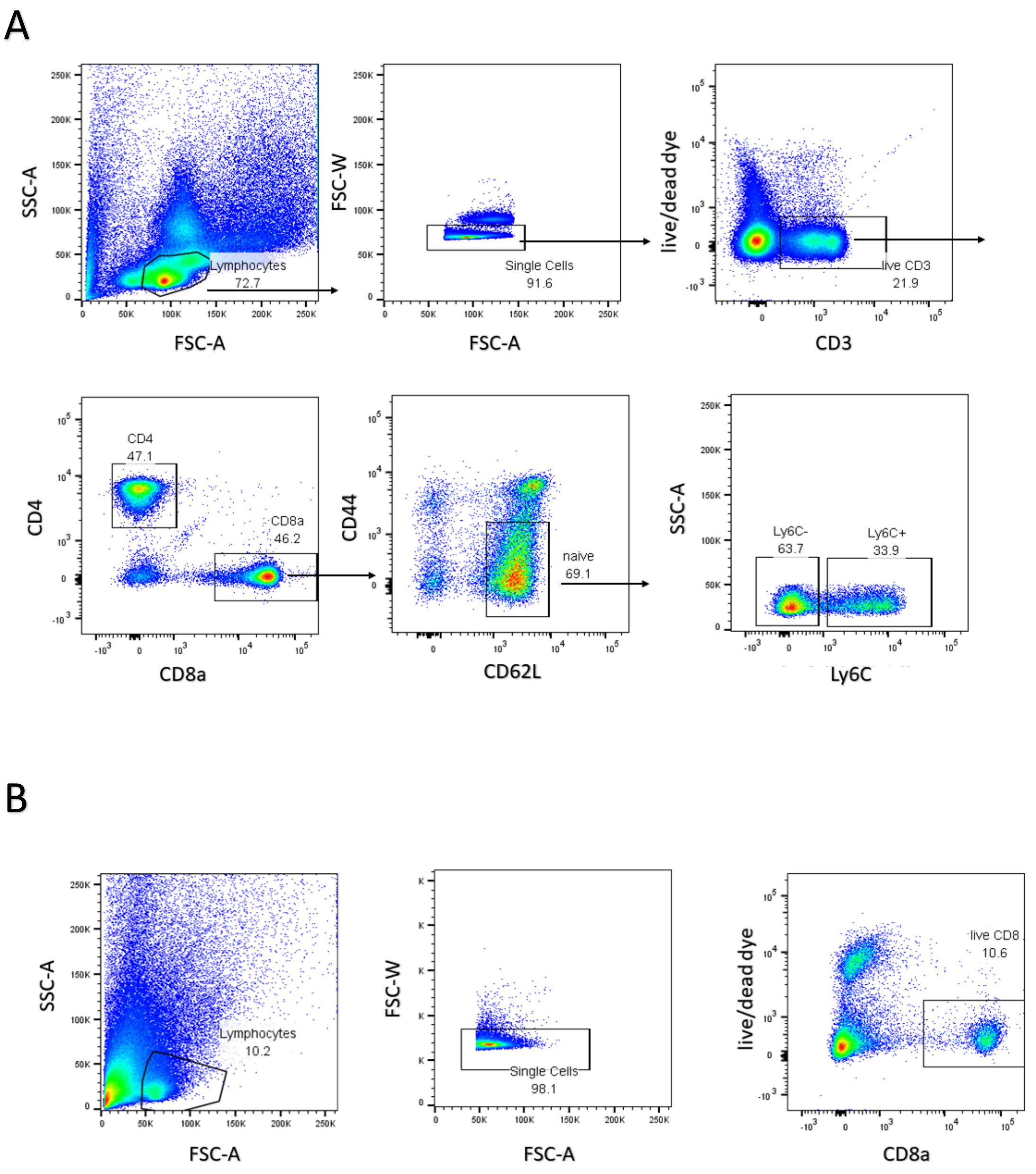

Supplemental Figure 7. Flow cytometric gating strategies. A) Gating strategy used to identify Ly6C positive and negative CD8^{+} Tn cells used in Figures 3A, 4B, 4D, 4E, 4G, 7C and for cell sorting. **B)** Gating strategy used to identify CD8 T cells transferred into RAG-KO mice depicted in Figure 7A. Identical gating strategy was used to analyze cultivated sorted Cd8 cells depicted in Figure 9D.

Antigen	Fluorochrome	Ab. clone	Manufacturer	Cat.No.	Dilution
CD3	BV750	17A2	Biolegend	100249	1/50
CD8a	BV785	53-6.7	Biolegend	100750	1/50
CD44	BV650	IM7	Biolegend	103049	1/50
CD62L	PEDazzle594	MEL-14	Biolegend	104448	1/50
Ly6C	BV570	HK1.4	Biolegend	128030	1/50
KLRG1	APCe780	2F1	ThermoFisher	47-5893-82	1/50
CD25	AF700	PC6.1	Biolegend	102024	1/50
CD4	BV711	GK1.5	Biolegend	100447	1/50
CD49d	FITC	RI-2	Biolegend	103606	1/100
CXCR3	PECy7	CXCR#173	Biolegend	126516	1/25
CCR7	PerCPCy5.5	4B12	ThermoFisher	45-1971-82	1/25
CD69	PE	H1.2F3	Biolegend	104508	1/50
CD122	efluor450	TM-S1	ThermoFisher	48-1222-82	1/50
GranzymeB	APC	QA16A02	Biolegend	372204	1/25
Tbet	PECy7	4B10	Biolegend	644824	1/50
IRF4	PE	IRF4.3E4	Biolegend	646404	1/25
Eomes	PerCP-eFluor 710	Dan11mag	ThermoFisher	46-4875-82	1/25
SCA-1	PECy7	D7	Biolegend	108114	1/50
CD5	PECy7	53-7.3	Biolegend	100622	1/50
pERK	AF488	20A	BD	612592	1/10
pZAP-70	PE	n3kobu5	ThermoFisher	12-9006-42	1/10
IFN-γ	AF700	XMG1.2	Biolegend	505824	1/25
TNF-α	e450	MP6-XT22	ThermoFisher	48-7321-82	1/25
BLIMP-1	PE	150006	Biolegend	150006	1/25
BCL-2	PECy7	BCL2/10C4	Biolegend	633512	1/25
BCL-6	PE/Dazzle™ 594	7D1	Biolegend	358510	1/25
MCL-1	PE	Y37	Biolegend	ab209289	1/100

Supplemental Table 1. List of antibodies used for flow cytometry analysis

Viruses	SPF	CH
MHV	0	83.3
MAD-2	0	50
MPV	0	16.6
MNV	0	6.25
**Bacteria**		
Mycoplasma pulmonis	0	83.3
Helicobacter sp.	0	100
**Parasites**		
Mites	0	100
Pinworms	0	100

Supplemental Table 2. Female C57BL/6 mice were cohoused with outbred, wild type pet shop mice in large rat cages, separated by a perforated barrier. After 3 weeks of cohousing C57BL/6 mice acquired multiple viral, bacterial and parasitic pathogens. The most consistently transmitted pathogens were external and internal parasites, mites and pinworms, which were present in all cohoused B6 mice. The most common viruses transmitted were the mouse hepatitis virus (MHV) and mouse adenovirus type II (MAD-2), and the majority of cohoused mice were also positive for Mycoplasma pulmonis
