# Supplementary material for: Infection-induced type I interferons critically modulate the homeostasis and function of CD8+ naïve T cells
Source: Nat Commun. 2021 Sep 6;12:5303. doi: 10.1038/s41467-021-25645-w (PMC8421345; doi:10.1038/s41467-021-25645-w)
Supplement: Supplementary file 3 — Reporting Summary [file 41467_2021_25645_MOESM3_ESM.pdf]

nature research

Corresponding author(s): Janko Nikolich-Zugich

Last updated by author(s): 08/05/2021

Reporting Summary

Nature Research wishes to improve the reproducibility of the work that we publish. This form provides structure for consistency and transparency in reporting. For further information on Nature Research policies, seeAuthors & Referees and theEditorial Policy Checklist .

Statistics

For all statistical analyses, confirm that the following items are present in the figure legend, table legend, main text, or Methods section.

n/a	Confirmed	
☐	☒	The exact sample size (*n*) for each experimental group/condition, given as a discrete number and unit of measurement
☐	☒	A statement on whether measurements were taken from distinct samples or whether the same sample was measured repeatedly
☐	☒	The statistical test(s) used AND whether they are one- or two-sided<br>*Only common tests should be described solely by name; describe more complex techniques in the Methods section.*
☐	☒	A description of all covariates tested
☐	☒	A description of any assumptions or corrections, such as tests of normality and adjustment for multiple comparisons
☐	☒	A full description of the statistical parameters including central tendency (e.g. means) or other basic estimates (e.g. regression coefficient) AND variation (e.g. standard deviation) or associated estimates of uncertainty (e.g. confidence intervals)
☐	☒	For null hypothesis testing, the test statistic (e.g. *F*, *t*, *r*) with confidence intervals, effect sizes, degrees of freedom and *P* value noted<br>*Give P values as exact values whenever suitable.*
☒	☐	For Bayesian analysis, information on the choice of priors and Markov chain Monte Carlo settings
☒	☐	For hierarchical and complex designs, identification of the appropriate level for tests and full reporting of outcomes
☒	☐	Estimates of effect sizes (e.g. Cohen's *d*, Pearson's *r*), indicating how they were calculated

Our web collection on statistics for biologists contains articles on many of the points above.

Software and code

Policy information about availability of computer code

Data collection	UAGC's Nextseq 500<br>FACS DIVA v 8.0.1.
Data analysis	Flowjo v10<br>Graphpad prism 7<br>SPSS Statistics 25<br>Cytobank Flowsom<br>DESeq2 R package<br>Tophat2 (v2.1.1)<br>HTSeq-count tool from the Python package HTSeq<br>Partex flow

For manuscripts utilizing custom algorithms or software that are central to the research but not yet described in published literature, software must be made available to editors/reviewers. We strongly encourage code deposition in a community repository (e.g. GitHub). See the Nature Research guidelines for submitting code & software for further information.

Data

Policy information about availability of data

All manuscripts must include a data availability statement. This statement should provide the following information, where applicable:

- Accession codes, unique identifiers, or web links for publicly available datasets
- A list of figures that have associated raw data
- A description of any restrictions on data availability

The data and materials that support the findings of this study are available from the corresponding author upon request. Source data are provided with this paper. The RNA-seq data discussed in Figure 5 of this publication have been deposited in NCBI's Gene Expression Omnibus and are accessible through GEO Series accession number GSE159076 [.https://www.ncbi.nlm.nih.gov/geo/query/acc.cgi?acc=GSE159076]. The flow cytometric data used for unbiased clustering in Figure 1 has been deposited to flow repository.org and is accessible through accession number FR-FCM-Z4AS [https://flowrepository.org/id/FR-FCM-Z4AS].

Field-specific reporting

Please select the one below that is the best fit for your research. If you are not sure, read the appropriate sections before making your selection.

☒ Life sciences ☐ Behavioural & social sciences ☐ Ecological, evolutionary & environmental sciences

For a reference copy of the document with all sections, see nature.com/documents/nr-reporting-summary-flat.pdf

Life sciences study design

All studies must disclose on these points even when the disclosure is negative.

Sample size	No sample size calulation was performed. Minimal sample size of N=4 was used due to cohousing limitations to IACUC protocol and to allow enough statistical power to detect differences between groups of mice.
Data exclusions	Mice were excluded from study only in the case of tumors or adverse reactions unrelated to the study design.
Replication	Each figure legend includes description of the number of replicates.
Randomization	Selection of laboratory animals for allocation to study groups was random.
Blinding	Blinding was not possible due to safety regulation in place, SPF and cohoused animals were kept in separate facilities and non SPF samples had to be processed in a BSL-3 laboratory. All mouse cages infected with West Nile Virus, Listeria Monocytogenes or cohoused with pet shop mice had lo be labeled as biohazard.

Reporting for specific materials, systems and methods

We require information from authors about some types of materials, experimental systems and methods used in many studies. Here, indicate whether each material, system or method listed is relevant to your study. If you are not sure if a list item applies to your research, read the appropriate section before selecting a response.

Materials & experimental systems

n/a	Involved in the study	
☐	☒	Antibodies
☐	☒	Eukaryotic cell lines
☒	☐	Palaeontology
☐	☒	Animals and other organisms
☒	☐	Human research participants
☒	☐	Clinical data

Methods

n/a	Involved in the study	
☒	☐	ChIP-seq
☐	☒	Flow cytometry
☒	☐	MRI-based neuroimaging

Antibodies

Antibodies used

Antigen Fluorochrome Ab. clone Manufacturer Cat.No. Dilution
CD3 BV750 17A2 Biolegend 100249 1/50
CD8a BV785 53-6.7 Biolegend 100750 1/50
CD44 BV650 IM7 Biolegend 103049 1/50
CD62L PEDazzle594 MEL-14 Biolegend 104448 1/50
Ly6C BV570 HK1.4 Biolegend 128030 1/50
KLRG1 APCe780 2F1 ThermoFisher 47-5893-82 1/50
CD25 AF700 PC6.1 Biolegend 102024 1/50

CD4 BV711 GK1.5 Biolegend 100447 1/50
CD49d FITC RI-2 Biolegend 103606 1/100
CXCR3 PECy7 CXCR#173 Biolegend 126516 1/25
CCR7 PerCPCy5.5 4B12 ThermoFisher 45-1971-82 1/25
CD69 PE H1.2F3 Biolegend 104508 1/50
CD122 efluor450 TM-S1 ThermoFisher 48-1222-82 1/50
GranzymeB APC QA16A02 Biolegend 372204 1/25
Tbet PECy7 4B10 Biolegend 644824 1/50
IRF4 PE IRF4.3E4 Biolegend 646404 1/25
Eomes PerCP-eFluor 710 Dan11mag ThermoFisher 46-4875-82 1/25
SCA-1 PECy7 D7 Biolegend 108114 1/50
CD5 PECy7 53-7.3 Biolegend 100622 1/50
pERK AF488 20A
612592 BD 612592 1/10
pZAP-70 PE n3kobu5 ThermoFisher 12-9006-42 1/10
IFN-γ AF700 XMG1.2 Biolegend 505824 1/25
TNF-α e450 MP6-XT22 ThermoFisher 48-7321-82 1/25
BLIMP-1 PE 150006 Biolegend 150006 1/25
BCL-2 PECy7 BCL2/10C4 Biolegend 633512 1/25
BCL-6 PE/Dazzle™ 594 7D1 Biolegend 358510 1/25
MCL-1 PE Y37 Biolegend ab209289 1/100

Validation

Antibodies were validated by the manufacturer. Manufacturers state:
Each lot of this antibody is quality control tested by immunofluorescent staining with flow cytometric analysis.

Eukaryotic cell lines

Policy information about cell lines

Cell line source(s)

L929 cells, Mouse fibroblast cell line, ATCC # CCL-1 CCL-1 is NCTC clone 929 of strain L, derived in 1948 from a C3H/An male mouse

Authentication

Describe the authentication procedures for each cell line used OR declare that none of the cell lines used were authenticated.

Mycoplasma contamination

Not tested for Mycoplasma contamination

Commonly misidentified lines
(See ICLAC register)

No commonly misidentified cell lines were used

Animals and other organisms

Policy information about studies involving animals; ARRIVE guidelines recommended for reporting animal research

Laboratory animals

Mice, C57BL/6, pet shop outbred mice, OT-1 TCR transgenic mice, IFNARKO mice, males and females, adults

Wild animals

No wild animals were used

Field-collected samples

No field-collected samples were used

Ethics oversight

Mouse studies were carried out in strict accordance with the recommendations in the Guide for the Care and Use of Laboratory Animals of the National Institutes of Health. Protocols were approved by the Institutional Animal Care and Use Committee at the University of Arizona (IACUC protocol 08-102, PHS Assurance No. A3248-01).

Note that full information on the approval of the study protocol must also be provided in the manuscript.

Flow Cytometry

Plots

Confirm that:

- [x] The axis labels state the marker and fluorochrome used (e.g. CD4-FITC).
- [x] The axis scales are clearly visible. Include numbers along axes only for bottom left plot of group (a 'group' is an analysis of identical markers).
- [x] All plots are contour plots with outliers or pseudocolor plots.
- [x] A numerical value for number of cells or percentage (with statistics) is provided.

Methodology

Sample preparation	Blood was collected into heparin-coated tubes and lysed hypotonically. Spleens and lymph nodes were mechanically dissociated through a 40 µm plastic mesh to prepare a single-cell suspension. Cells were stained with surface antibodies, and then fixed and permeabilized using the FoxP3 Fix/Perm kit (eBioscience, San Diego, CA) for intracellular staining.
Instrument	BD LSR Fortessa or BD FACS Aria III
Software	Flowjo v10
Cell population abundance	A mimimum of 20000 CD8+ T cells collected per sample
Gating strategy	Lymphocytes were identified by size and granularity in the FSC/SSC scatter. Dead cells were excluded using live/dead fixable dyes. Borders of positivity and negativity were identified using FMO controls. Median fluorescence intensity was used to determine expression.

☒ Tick this box to confirm that a figure exemplifying the gating strategy is provided in the Supplementary Information.
